# KLF4 represses MAP2K7 signaling in T-ALL

**DOI:** 10.18632/oncotarget.20672

**Published:** 2017-09-06

**Authors:** H. Daniel Lacorazza

**Affiliations:** H. Daniel Lacorazza: Department of Pathology & Immunology, Baylor College of Medicine, Texas Children’s Hospital, Houston, Texas, USA

**Keywords:** T-ALL, KLF4, NOTCH1, JNK, LIC

A major challenge in the therapy of cancer patients is the development of novel drugs that efficiently target cancerinogenic pathways and replace the highly toxic chemodrugs developed over 50 years ago that non-specifically eliminate highly proliferative cells. The main goal of discovering new cancer specific drugs is not only to improve the treatment response and cure rate but also to prevent the devastating side effects of traditional chemodrugs. This requires refocusing research efforts toward the basic aspects of the pathobiology of cancer and the identification of new molecular targets.

**Figure 1:**
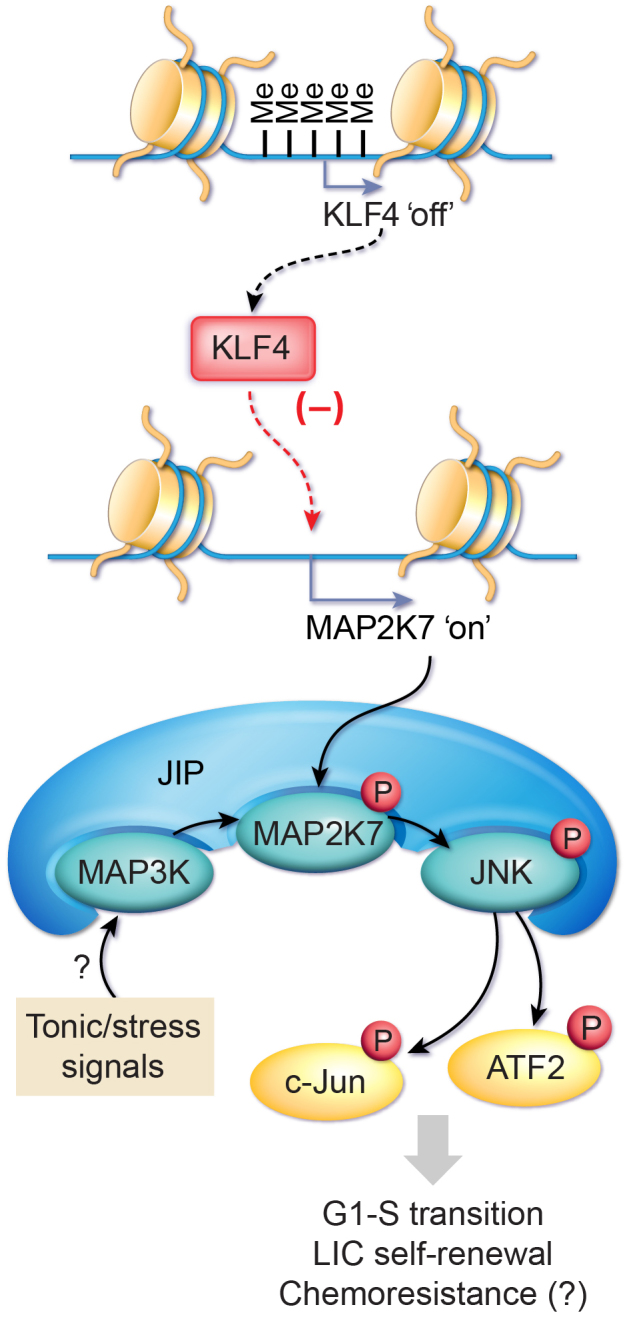
Diagram depicting the tumor suppressive role of KLF4 in pediatric T-ALL.

The model of the cancer stem cell has gained significant attention recently because this rare cell population is believed to drive chemoresistance and relapses. Despite improvements in risk-adaptive chemotherapy using multiple agents in T cell acute lymphoblastic leukemia (T-ALL), relapse remains the leading cause of cancer-related death in children, with high-risk T-ALL patients in desperate need of an alternative targeted therapy to eliminate leukemia-initiating cells (LIC) [1, 2]. Our group recently reported a novel tumor suppression function for the transcription factor KLF4 in pediatric T-ALL [3, 4], which is best known for reprogramming somatic cells to pluripotent stem cells. We show that KLF4 transcripts are significantly downregulated in samples from children with T-ALL, especially in patients with a poor prognosis, such as the ETP-ALL and TLX groups [3, 5]. Low levels of KLF4 expression correlate with increased CpG methylation (Me) at the proximal promoter (Figure 1), which is confirmed by monitoring the re-expression of KLF4 after treating T-ALL cell lines with a demethylating agent. Although classical tumor suppressors are commonly mutated in cancer patients, to date, only one report describes mutations in the KLF4 gene in T-ALL patients [6]. Nonetheless, the regulation of KLF4 expression by epigenetic mechanisms, such as DNA methylation, is described in several cancers. This is consistent with an emerging paradigm postulating that the epigenome contributes to the inactivation of tumor suppressors and clonal heterogeneity in addition to recurring chromosomal abnormalities and gene mutations.

The loss-of-function mouse model of NOTCH1-induced T-ALL clearly shows that the genetic deletion of KLF4 leads to a more aggressive disease with a shorter latency and higher penetrance [3]. Strikingly, a hemizygous deletion of the KLF4 gene displays an acceleration of the disease induced by NOTCH1 similar to the homozygous deletion because the heterozygous T-ALL cells display low levels of KLF4 expression that are closer to the homozygous T-ALL cells. A molecular analysis reveals that the rapid development of T-ALL is associated with an increased transition from the G1- to S-phase in bulk leukemic cells, an expansion of LICs (increased self-renewal) determined by limiting-dose transplantation and immunophenotypic enumeration, and the repression of the *MAP2K7* gene through analysis of gene expression array and chromatin-immunoprecipitation and sequencing. Consistent with the KLF4 repression of *MAP2K7*, the genetic (mouse model) or epigenetic (patients) loss-of-KLF4 expression is associated with elevated levels of the MAP2K7 protein. However, the aberrant activation of the MAP2K7 signaling (phosphorylated MAP2K7 and downstream targets JNK, c-JUN, ATF2) in both leukemia blasts and LICs suggests a basal activation of MAP2K7 (Figure 1; JIP, JNK interacting protein). Furthermore, data mining of the gene expression profiles in T-ALL patients versus healthy children and the ectopic expression of KLF4 in T-ALL cell lines suggests an inverse correlation of MAP2K7 activation and KLF4 expression.

Although the discovery of the KLF4-MAP2K7-JNK pathway in T-ALL is still in its early stages for future translation to patients, it provides a novel target to develop new LIC-specific therapies. It is important to establish first, in a large cohort of patients, whether the expression of KLF4 and MAP2K7 stratifies high-risk patients for the inhibition of MAP2K7 signaling as an adjuvant to standard multidrug therapy, with the goal of increasing the treatment response and diminishing the incidence of relapse. The use of JNK inhibitors is an attractive approach for therapy despite its poor specificity, potential cellular toxicity, and activity at micromolar concentrations [7]. We evaluated the efficacy of ATP-competitor JNK inhibitors tested in clinical trials, such as CC-401 (Phase I) and orally active AS602801 (Phase II), in cell lines and patient-derived xenografts both *in vitro* and *in vivo* as a proof-of-concept to validate our model and support the development of alternative therapies for relapse T-ALL. Hence, more research is needed to test direct inhibition of MAP2K7 and to identify the mechanism underlying the activation of MAP2K7 in LICs perhaps through metabolic and genomic stress or alternatively through tonic signals emanating from faulty cell surface receptors (Figure 1). These tonic signals could maintain the leukemic stem cell population that feeds this neoplasm; thus, their inhibition would allow targeting LICs.

Pharmacological inhibition of cooperative events rather than initial genetic drivers, such as NOTCH1, will likely be more efficient for targeting the heterogeneity of leukemia generated during the cancer clonal evolution in relapsed patients. For example, KLF4 is expressed in resistant clones in chronic-lymphoblastic leukemia [8]. Our findings shed light on a novel mechanism of T-ALL suppression by preventing the expansion of LIC. This information could lead to the development of the first LIC-targeted therapy for T-ALL aimed at reducing the toxicities associated with multi-drug chemotherapy and, most importantly, improving the survival of patients with refractory and relapsed disease.
